# An Automated Platform for Assessment of Congenital and Drug-Induced Arrhythmia with hiPSC-Derived Cardiomyocytes

**DOI:** 10.3389/fphys.2017.00766

**Published:** 2017-10-11

**Authors:** Wesley L. McKeithan, Alex Savchenko, Michael S. Yu, Fabio Cerignoli, Arne A. N. Bruyneel, Jeffery H. Price, Alexandre R. Colas, Evan W. Miller, John R. Cashman, Mark Mercola

**Affiliations:** ^1^Department of Medicine, Cardiovascular Institute, Stanford University, Stanford, CA, United States; ^2^Graduate School of Biomedical Sciences, Sanford Burnham Prebys Medical Discovery Institute, La Jolla, CA, United States; ^3^Sanford Burnham Prebys Medical Discovery Institute, La Jolla, CA, United States; ^4^Department of Bioengineering, University of California, San Diego, San Diego, CA, United States; ^5^Vala Sciences, San Diego, CA, United States; ^6^Departments of Chemistry, Molecular and Cell Biology, Helen Wills Neuroscience, University of California, Berkeley, Berkeley, CA, United States; ^7^Human BioMolecular Research Institute, San Diego, CA, United States

**Keywords:** induced pluripotent stem cells, cardiomyocyte, voltage sensitive probe, high throughput screening, drug development, cardiotoxicity, CiPA

## Abstract

The ability to produce unlimited numbers of human induced pluripotent stem cell derived cardiomyocytes (hiPSC-CMs) harboring disease and patient-specific gene variants creates a new paradigm for modeling congenital heart diseases (CHDs) and predicting proarrhythmic liabilities of drug candidates. However, a major roadblock to implementing hiPSC-CM technology in drug discovery is that conventional methods for monitoring action potential (AP) kinetics and arrhythmia phenotypes *in vitro* have been too costly or technically challenging to execute in high throughput. Herein, we describe the first large-scale, fully automated and statistically robust analysis of AP kinetics and drug-induced proarrhythmia in hiPSC-CMs. The platform combines the optical recording of a small molecule fluorescent voltage sensing probe (VoltageFluor2.1.Cl), an automated high throughput microscope and automated image analysis to rapidly generate physiological measurements of cardiomyocytes (CMs). The technique can be readily adapted on any high content imager to study hiPSC-CM physiology and predict the proarrhythmic effects of drug candidates.

## Introduction

Between 1991 and 2003, 6 drugs were withdrawn from the market in the U.S. because of evidence that they induced QT prolongation and the potentially fatal ventricular tachycardia (VT), Torsade de Pointes (TdP) (Waring et al., 2015). At a cellular level, drug-induced VT is precipitated most commonly by drug inhibition of the human ether-à-go-go (hERG) channel. hERG conducts the rapidly activating component of the delayed rectifier potassium current (I_Kr_) that is responsible for the bulk of ventricular repolarization. Following regulatory agency guidelines, the *in vitro* measurement of hERG inhibition has become essential for advancement of a drug candidate to clinical development (Waring et al., 2015). While the results of hERG inhibition studies have kept dangerous drugs from reaching the market, it is poorly predictive of the development of TdP. Many drugs can inhibit hERG without causing VT or TdP (Redfern et al., 2003; Kramer et al., 2013) and some drugs can cause VT or TdP by other mechanisms (Roden, 1998; Lacerda et al., 2008). Moreover, hERG inhibition is highly sensitive to a wide range of chemical substructures (Sanguinetti and Tristani-Firouzi, 2006) that might comprise clinically beneficial components of small molecule drugs. There is considerable concern that reliance on hERG inhibition alone may prevent many useful compounds from advancing toward the clinic. This concern was embodied in the 2013 Food and Drug Administration recognition of the need for a comprehensive non-clinical assay to better predict the proarrhythmic potential of new drugs (Comprehensive *in vitro* Proarrhythmia Assay (CiPA) initiative) (Sager et al., 2014).

hiPSC-CMs are an emerging model to advance the field beyond using hERG or other single ion channels in heterologous expression systems as an *in vitro* tool to predict arrhythmogenic potential (Liang et al., 2013; Mercola et al., 2013). Other studies have shown that hiPSC-CMs express most of the ion channels of ventricular CMs and can be produced in limitless quantities from normal individuals as well as from patients with arrhythmia proclivity (Ma et al., 2011). This unique property creates an unprecedented opportunity to gain insight into drug responses on a range of genetically diverse individuals during the early stages of drug development. In addition, hiPSC-CMs make it possible to determine if certain naturally occurring polymorphisms in ion channels, discovered by genome wide association studies (GWAS) or incidentally by DNA sequencing, increase susceptibility to adverse drug responses.

To accommodate the demand to rapidly and quantitatively assess preclinical candidates through dose ranges and to evaluate genetically diverse hiPSC-CM models, we developed an automated platform to optically assess AP kinetics and quantify cellular arrhythmia phenotypes. The 384-well format assay described herein combines a small molecule voltage sensitive probe (VSP), VoltageFluor2.1.Cl (VF2.1.Cl) (Miller et al., 2012), and an automated high speed/high resolution microscope to generate AP kinetic measurements from optical recordings of CMs. Initially, we validated the assay conditions using primary rat atrial and ventricular CMs, and visualized chamber-specific drug effects. Next, we characterized the functionality of voltage-gated ion channels and β-adrenergic receptors expressed on the membrane of hiPSC-CMs with a panel of reference compounds. Furthermore, using molecules known to inhibit hERG and cause clinically relevant arrhythmia, we observed a dose-dependent drug induction of early after depolarizations (EADs) that are cellular manifestations of T-wave prolongation and premature ventricular contractions (PVCs) on the electrocardiogram (ECG). Finally, using a genetic model of congenital Long QT syndrome type 3 (LQTS3) and pharmacological models of LQTS2 and LQTS3, both of which predispose patients to VT and sudden death, we demonstrated the reversion of these disease phenotypes in a 384-well multi-well format assay. We conclude that the screening platform described herein makes it possible to comprehensively evaluate the proarrhythmic potential of many compounds in hiPSC-CMs derived from multiple patients, demonstrating the feasibility of utilizing hiPSC-CMs and physiological screening during early stage drug development.

## Materials and methods

### Isolation of rat cardiomyocytes

Neonatal atrial and ventricular rat cardiomyocytes were isolated with the neonatal rat cardiomyocyte isolation kit (Worthington, New Jersey, USA) (Toraason et al., 1989; Macgregor et al., 1995) and cultured at 37°C with 5% CO_2_. Briefly, heart atria and ventricles were dissected from 1 day old Hsd:Sprague–Dawley rats, then digested overnight at 4°C with trypsin. Digestion continued 12 h later with collagenase for approximately 90 min at 37°C. Isolated cells were pre-plated for 120 min on uncoated cell culture dishes to remove fibroblasts, and subsequently, atrial and ventricular cardiomyocytes were either pooled or separately seeded on Matrigel-coated 96-Well Glass-Bottom Plates (SensoPlate™, Greiner Bio-One, North Carolina, USA) in high-serum media [DMEM/F12 (1:1), 0.2% bovine serum albumin, 3 mM sodium-pyruvate, 0.1 mM ascorbic acid, 4 mg l21 transferrin, 2 mM L-glutamine, 100 nM thyroid hormone (T3) supplemented with 10% horse serum and 5% fetal calf serum (FCS)] at a density of 4 × 10^5^ cells/well. After 24 h, media was changed to low-serum medium (the same as above but substituting 0.25% FCS for 10% horse serum and 5% FCS). Seventy two hours after seeding the cells, they were processed for voltage imaging. All animal handling and care followed the NIH Guide for Care and Use of Laboratory Animals. The experimental protocols were approved by Institutional Animal Care and Use Committees of Sanford-Burnham-Prebys Medical Discovery Institute and Stanford University.

### Culture of hiPSC-CMs

Vials of both MyCell (LQTS3) and iCell (healthy individual) Cardiomyocytes (Cellular Dynamics International, Wisconsin, USA) were thawed according to the manufacturer's specifications (see Supplementary Table 1 for lot information). A single cryovial was removed from liquid nitrogen and placed on dry ice for no more than 3 min. The vial was transferred to a 37°C water bath and incubated for 4 min. Once completely thawed, the contents were slowly transferred to a 50 mL conical vial using a 1,000 μL pipette. 1 mL of iCell Cardiomyocyte Plating Media (iCCPM) was used to rinse the inside of the cryovial to gather any remaining cells. After rinsing the vial, the 1 mL of iCCPM was added dropwise to the 50 mL conical vial over a period of 2–3 min with continuous agitation for each drop. The additional volume for dilution was calculated to achieve a final concentration of 2.5 × 10^5^ cells/mL and depended on the specifications of each lot of cells. The additional media was added dropwise to the 50 mL vial. Once diluted, the cells were transferred into a reagent reservoir and 20 μL was added to each well with a 16 channel pipette (Integra Viaflo II) at a flow rate of 35 μL/s with 1 mix cycle prior to each addition in a pre-coated 384 well plate (Greiner Bio-One) with 0.1% (w/v) gelatin (Stem Cell Technologies). The plates were left standing at room temperature for 20 min to allow the cells to settle to the bottom of the plate before it was placed in a 37°C 5% CO_2_ incubator. After 24 h, the plating media was diluted by adding 80 μL of iCell Cardiomyocyte Maintenance Media (iCCMM), supplemented with 5 mM D-glucose, and subsequently 50 μL of iCCMM was removed are replaced with 50 μL of iCCMM. This process was repeated two additional times, ultimately yielding a final volume of 100 μL/well. The plates were placed back in the 37°C 5% CO_2_ incubator for 48 h. Media was exchanged every other day for 14 days prior to imaging by removing 50 μL of media and adding 50 μL of fresh iCCMM.

### Differentiation of hiPSCs to cardiomyocytes

Briefly, hiPSCs (hiPSC line SCVI15; see Supplementary Table 1) were dissociated using 0.5 mM EDTA (ThermoFisher Scientific) in PBS without CaCl_2_ or MgCl_2_ (Corning) for 7 min at room temperature. The dissociated hiPSCs were plated at a density of 3 × 10^5^ cells per well of a Matrigel-coated 12 well plate in mTeSR1 media (StemCell Technologies) supplemented with 2 μM Thiazovivin (Selleck Chemicals). After 24 h, the media was replaced with mTESR1 without Thiazovivin and was replenished daily for 3–5 days until the cells reached ≥90% confluence to begin differentiation. Cardiomyocytes were differentiated by methods previously described (Burridge et al., 2014; Cunningham et al., 2017). At day 25, cells were dissociated and plated onto Matrigel-coated 384-well tissue culture plates (Greiner Bio-One) at a density of 20,000 cells/well. All experiments were performed day 28 post-differentiation.

### Preparation of VF2.1.Cl loading solution and reference compounds

VF2.1.Cl (the dye used in this study was synthesized by EWM, but is marketed as Fluovolt) dye loading solution and the compound dilutions were prepared prior to manipulation of the cells on the day of imaging. 1 μL of 2 mM VF2.1.Cl in DMSO was mixed with 1 μL of 10% Pluronic F127 (diluted in water) in a 1.7 mL tube by agitating and centrifuging 3 times. Mixing of VF2.1.Cl with Pluronic F127 prior to subsequent dilution in physiological buffer is critical for VF2.1.Cl loading. Separately, Hoechst 33258 was diluted into Tyrode's solution (136 mM NaCl, 5 mM KCl, 2 mM CaCl_2_, 1 mM MgCl_2_, 10 mM glucose, 10 mM HEPES, pH 7.4) to a concentration of 4 μg/mL. 1 mL of the Hoechst/Tyrode's solution was added to the 1.7 mL tube containing the VF2.1.Cl/Pluronic F127 mixture and vortexed for 10 s. The contents of the 1.7 mL tube was added to 4 mL of Hoechst/Tyrode's solution. The mixture (5 mL of a 2x concentrated VF2.1.Cl dye loading solution) can be scaled to larger volumes if reagent ratios are maintained. Reference compounds isoproterenol (Tocris, Bristol, UK), propranolol (Tocris, Bristol, UK), verapamil (Tocris, Bristol, UK), tetrodotoxin (Tocris, Bristol, UK), ATX-II (Sigma Aldrich, Missouri, USA), mexiletine (Toronto Research Chemical, Toronto, Canada), ranolazine (Tocris, Bristol, UK), acetylcholine (Sigma Aldrich, Missouri, USA) and sotalol (Tocris, Bristol, UK) were diluted in water and formoterol (Tocris, Bristol, UK), isradipine (Tocris, Bristol, UK), BayK 8644 (Tocris, Bristol, UK), veratridine (Tocris, Bristol, UK), E-4031 (Tocris, Bristol, UK), and dofetilide (Tocris, Bristol, UK) were diluted in DMSO. Each compound was diluted in Tyrode's solution to a 2x concentrated stock and was warmed to 37°C using a dry heat block prior to application to the cells.

### Loading of VF2.1.Cl dye solution and automated image acquisition

hiPSC-CMs were removed from a 37°C 5% CO_2_ incubator and placed immediately into in a tissue culture cabinet on a dry heat block set to 37°C to prevent temperature fluctuation during the subsequent washing and dye loading steps. All subsequent manipulations were conducted on a 37°C dry heat block. Tyrode's solution was warmed to 37°C in a water bath. To wash the cells and remove the media, 50 μL of media was removed and replaced with 50 μL of Tyrode's solution five times using a 16 channel pipette at flow rate of 35 μL/s. After the fifth wash, 50 μL of the 2x VF2.1.Cl dye loading solution was added to each well. The plate was placed back in the 37°C 5% CO_2_ incubator for 50 min. After the 50 min incubation, the cells were rinsed 4 more times by removing 50 μL of Tyrode/dye solution from the well and adding 50 μL of fresh Tyrode's solution to the well and repeating. After rinsing, the cells were placed back in a 37°C 5% CO_2_ incubator for 10 min to recover. After recovery, 50 μL of solution was removed and 50 μL of 2x reference compound was added to the respective well and incubated at 37°C and 5% CO_2_ for 5 min before image acquisition. Time series images were acquired automatically using the IC200 KIC instrument (Vala Sciences, California, USA) at an acquisition frequency of 100 Hz for a duration of 6.5 s or 33 Hz for 20 s, as indicated in the figure legends, with excitation wavelength of 485/20 nm and emission filter 525/30 nm using a 0.75 NA 20x Nikon Apo VC objective. A single image of the Hoechst was acquired after the time series. The optimized dye loading and imaging conditions were replicated using both a different high content imager, the ImageXpress Micro XLS platform (Molecular Devices), and hiPSC-CMs produced by a novel differentiation protocol (Cunningham et al., 2017; and Supplementary Figure 3).

### Image analysis, physiological parameter calculation, and data analysis

The image analysis and physiological parameter calculation was conducted using commercially available Cyteseer (Vala Sciences) as previously described (Cerignoli et al., 2012; Lu et al., 2015). Briefly, the image output by Cyteseer Scanner were loaded into Cyteseer and the whole well cardiac time series algorithm was executed on the image files. The metrics analyzed by the whole well cardiac time series algorithm were beat rate, normalized area under the peak trace (normalized peak integral), and APD_25_, APD_50_, APD_75_, and APD_90_ (referring to the width of the cardiac AP at a point 25, 50, 75, and 90% respectively), from the peak to baseline amplitude. EADs were quantified automatically using the default noise threshold in the whole well cardiac time series algorithm. The algorithm determines EADs as peaks following a local minimum at a user-specified threshold above the diastolic interval minimum. Data tables were analyzed using Microsoft Excel 2013 and dose response curves and heat maps were calculated using GraphPad Prism 7 software.

### Immunohistochemistry

After live imaging using VF2.1.Cl, the neonatal CMs were fixed using 4% paraformaldehyde. After fixation, the cells were incubated with a blocking solution [10% horse serum, 0.5% Triton X-100, 0.01% gelatin in phosphate buffered saline (PBS)] for 30 min at room temperature followed by incubation with anti-Myl2 (Proteintech, cat: 10906-1-AP) or anti-Myl7 (Synaptic Systems, cat: 311 011) for 1 h at room temperature. Following incubation with the primary antibody, the cells were washed 5 times with PBS before incubation with anti-mouse Alexa Fluor 568 (ThermoFisher Scientific, A-11057) or anti-rabbit Alexa Fluor 647 (ThermoFisher Scientific, A-31573) for 1 h at room temperature. After incubation with the secondary antibody, the cells were washed 5 times with PBS and stored in a 50% glycerol (v/v) solution in PBS for subsequent imaging using the KIC.

### Individual cell image analysis

The single cell analysis was performed manually using ImageJ. Regions of interest (ROIs) were selected using the freehand selection tool and exported as .roi files. Using nuclear labeling from the live and fixed images, offsets for the image sets were manually determined. The ROIs were then applied to the time series images and used to generate fluorescent intensity values for each ROI in all images. The data was analyzed using Microsoft Excel 2013 and plotted using GraphPad Prism 7.

### Meta-analysis of experimental data

An R-script (R version 3.3.1) was developed that reads the CSV files generated by Cyteseer and amalgamates all the physiological parameters of interest into a single file. Using this dataset, we performed a meta-analysis of all experiments and determined the intra and inter thaw variability in APD_75_ as well as the relationship between APD_75_ and the beat rate. As a result of the non-linear relationship of APD_75_ and the beat rate, statistical significance was determined using a sliding window (center value ± 3) to mirror experimental ranges and applying the Mann-Whitney test (using the Bonferroni criterion for multiple testing).

### Whole cell patch clamp electrophysiology

Cardiac ion currents were recorded from single cardiomyocytes using the whole-cell patch-clamp method. Briefly, coverslips with cardiomyocytes were transferred into electrophysiological perfused recording chamber (RC-25-F, Warner Instruments, Hamden, CT) mounted on the stage of an inverted Olympus microscope. Patch pipettes were pulled from thin-wall borosilicate glass capillaries (CORNING 7740, 1.65 mm) with a P-2000 laser pipette puller (Sutter Instruments, California, USA) and had electrode tip resistances between 1.5 and 5.5 MΩ with access resistance of < 8 MΩ for whole-cell patch recordings. Series resistance and cell capacitance were compensated to between 30 and 60% in some voltage-clamp recordings. Patch electrodes were filled with intracellular solution containing: 120 mM CsCl, 20 mM tetraethylammonium chloride (TEA-Cl), 10 mM Hepes, 2.25 mM EGTA, 1 mM CaCl_2_, 2 mM MgCl_2_, pH 7.4. All recordings were performed at room temperature in Tyrode's solution. Current response traces were acquired using the Axon 200B amplifier. Currents were digitally sampled at 10 kHz using Digidata 1322A digitizer hardware and pClamp 10.2 software (Molecular Devices, California, USA). Current responses were filtered using 8-pole Bessel analog low-pass filter at 1-2 kHz cutoff frequency. In order to record the late sodium component, the pipette-cell seal stability and access resistance was carefully monitored during the recording. Current amplitude at the end of the depolarizing step was corrected by the leak current and the corrected current amplitude was averaged for 10 cells. After the leak-correction, we computed the average value of the recorded current amplitude over the last 50 ms of the 150 ms depolarizing pulse of each 250 ms sweep and averaged the resulting value over 3 consecutive sweeps.

## Results

### Automated acquisition of action potential kinetics with VF2.1.Cl

**Figure 1:**
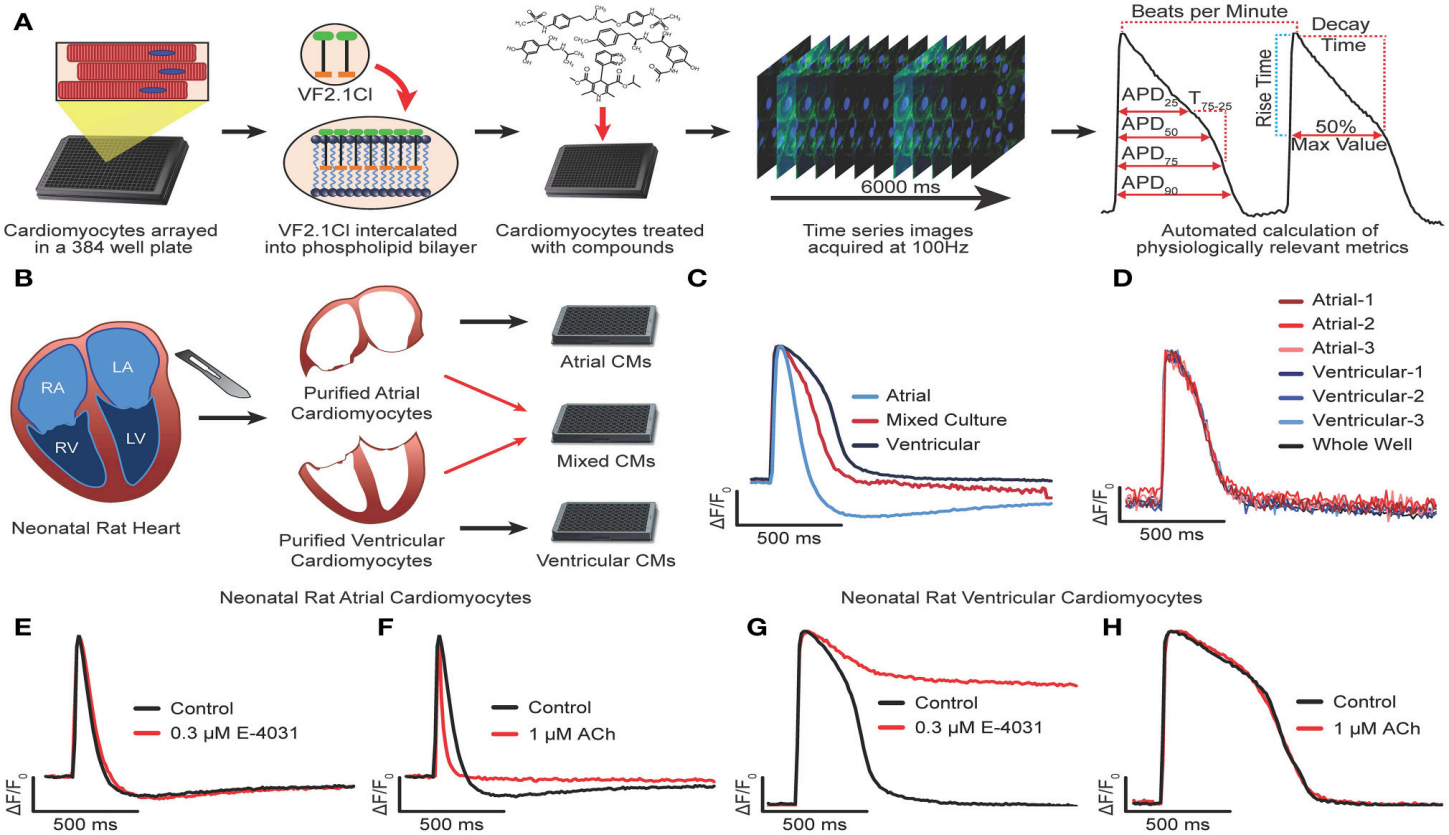
Development and optimization of high throughput optical recording of VF2.1.Cl in cardiomyocytes. **(A)** Schematic of workflow for HTS transmembrane potential assay. VF2.1.Cl, VoltageFluor2.1.Cl voltage sensitive dye. **(B)** Schematic of workflow to isolate neonatal rat CMs from the atria and the ventricles and plated either in isolation or as a 1:1 mixture. **(C)** Normalized ΔF/F_0_ vs. time plots (100 Hz) generated from wells containing isolated neonatal rat atrial CMs, isolated ventricular CMs or a mixed culture of rat atrial and ventricular CMs paced by electric field stimulation at 0.3 Hz. **(D)** Overlay of normalized ΔF/F_0_ vs. time plots generated from individual clusters atrial and ventricular CMs as well as from the whole well average ΔF/F_0_ vs. time. **(E–H)** Normalized ΔF/F_0_ vs. time plots for isolated atrial **(E,F)** or ventricular **(G,H)** CMs treated with 0.3 μM E-4031 **(E,G)**, 1 μM acetylcholine **(F,H)** as compared to an untreated well. Scale bars indicate time (500 ms) and ΔF/F_0_ (25% of the maximal obtained in each well).

To create the automated platform, we developed methods for high throughput acquisition of high speed/high resolution (5.5 megapixel at 100 Hz) time series images as well as downstream image analysis to calculate physiological parameters that describe the AP kinetics of CMs in electrical syncytium (Figure 1A). To measure changes in membrane potential, the platform utilized VF2.1.Cl (Miller et al., 2012), which intercalates into the plasma membrane and senses changes in transmembrane potential by a novel photo-induced electron transfer (PeT) mechanism (Miller, 2016; VF2.1.Cl and related VSPs have been described in detail Woodford et al., 2015; Deal et al., 2016; Kulkarni et al., 2017, and can be obtained directly from EWM). VF2.1.Cl and structurally related probes generate a larger change in fluorescence in response to changes in voltage than conventional electrochromic dyes while maintaining sufficient time resolution to measure cardiomyocyte APs (Miller et al., 2012; Supplementary Figure 1A). We established a robust protocol to standardize cell culture conditions that consistently achieved synchronously contracting monolayers of hiPSC-CMs as well as dye loading and imaging conditions that produced optical traces with sufficient signal-to-noise and reproducibility (Supplementary Movie 1). 200 nM VF2.1.Cl and ~120 mW/cm^2^ illumination intensity (excitation: 485/20 nm, emission: 525/30 nm) enabled us to achieve excellent signal-to-noise, while maintaining reproducibility (Supplementary Figure 1B). Excessive illumination or VF2.1.Cl concentration caused variability in the AP response manifested by a prolongation of the APD within successive peaks from a single recording of an individual well (Supplementary Figures 1C–K). This observation is consistent with previous reports for the VSP, di-4-ANEPPS (Schaffer et al., 1994). Thus, our optimization provided conditions for statistically robust analysis of synchronously contracting monolayers of hiPSC-CMs for high throughput screening applications.

We began development of our AP waveform analysis procedures using bona fide ventricular and atrial cardiomyocytes isolated from the neonatal rat heart (Figures 1B–H). Purified atrial and ventricular cardiomyocyte populations, cultured as an electrically coupled monolayer, exhibited characteristic AP morphologies using a whole well analysis (Figure 1C). However, chamber specific morphologies could not be discerned when atrial and ventricular cardiomyocytes were co-cultured in a 1:1 ratio, either at the whole well analysis, or by analyzing the waveforms of individual Myl2^+^ (ventricular) or Myl7^+^ (atrial) cardiomyocytes identified retrospectively (see Supplementary Figure 1L and Methods for overlaying waveform and immunostaining). Instead, the individual waveforms and whole well waveforms were indistinguishable (Figure 1D). In addition, no difference in AP morphology was observed in the whole well vs. individual cell waveforms of the hiPSC-CM cultures (Supplementary Figures 1M,N). These results show that AP morphology acquired from the whole well ROI represents that of all individual CMs, regardless of subtype identity, in a synchronously contracting monolayer. This result suggests that an effect on one cell type (i.e., atrial) might be masked by an opposing effect on another (i.e., ventricular) in an electrical syncytium of multiple cell types. However, whole well analysis greatly reduced the computational workload when compared to single cell analysis and enabled the rapid generation of physiological parameters. Using this approach, we could visualize characteristic atrial specific drug effects in pure populations of neonatal rat atrial and ventricular cardiomyocytes. Acetylcholine, a selective agonist of I_Ach_ shortens the APD in atrial but not ventricular myocytes (Wang et al., 1993; Koumi et al., 1994; Devalla et al., 2015) whereas E-4031, a selective blocker of I_Kr_ mediated by hERG, prolonged APD in ventricular CMs but not atrial CMs (Figures 1E–I). Atrial CMs utilize IK_ur_ mediated by K_v_1.5 in addition to IK_r_ for repolarization, and are less sensitive hERG block (Roy et al., 1996). Thus, high throughput optical recording of VF2.1.Cl and whole well analysis was sufficient to distinguish chamber specific drug effects.

### Characterization of hiPSC-CMs with reference compounds

**Figure 2:**
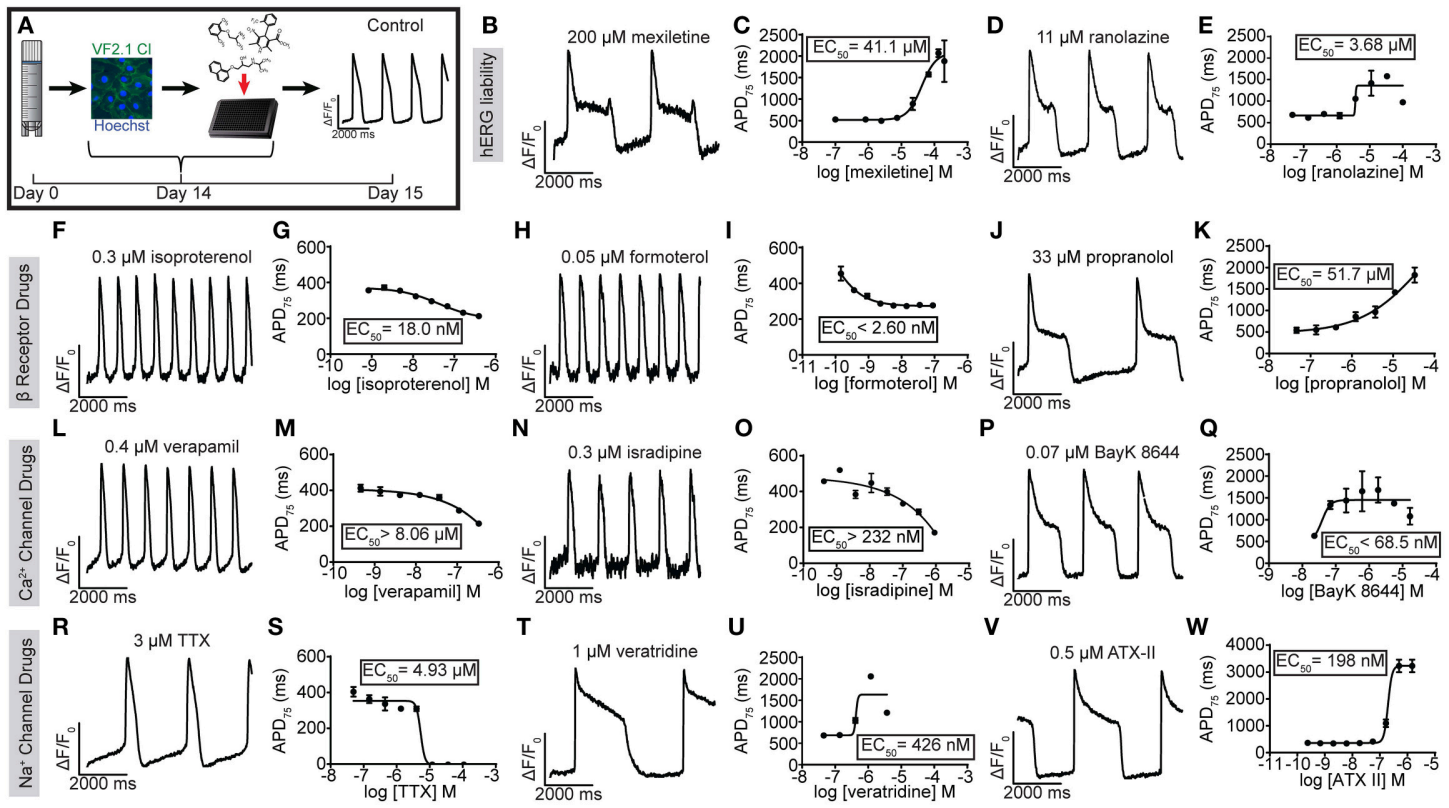
Utility of VF2.1.Cl in measuring the effect of reference compounds on hiPSC-CM AP kinetics. **(A)** Schematic of treatment of normal individual (iCell) hiPSC-CMs with reference compounds and a ΔF/F_0_ vs. time plot of an untreated well. **(F–W)** Representative normalized ΔF/F_0_ vs. time plot (100 Hz) and dose response curve for APD_75_ for mexiletine **(B,C)**, ranolazine **(D,E)** isoproterenol **(F,G)**, formoterol **(H,I)**, propranolol **(J,K)**, verapamil **(L,M)**, isradipine **(N,O)**, BayK 8644 **(P,Q)**, TTX **(R,S)**, veratridine **(T,U)**, and ATX-II **(V,W)** in hiPSC-CMs. Each point represents an individual well, *n* = 3. Error bars are s.e.m. Scale bars indicate time (2,000 ms) and ΔF/F_0_ (50% of the maximal obtained in each well).

The ability of hiPSC-CMs to recapitulate drug effects observed in adult CMs is critical for their use in both disease modeling and proarrhythmia evaluation. Therefore, we evaluated the expression and functionality of ion channels and adrenergic receptors with a panel of reference compounds (Figure 2A). To calculate EC_50_ values, the cells were acutely treated with each compound (*n* = 3 wells per dose) and the average whole well fluorescent intensity was plotted and the waveforms were used to calculate the APD_75_ for each well. Mexiletine and ranolazine are known to have an off-target inhibition of hERG, and indeed, the compounds caused a dose dependent APD prolongation and EADs at doses similar to those reported (EC_50_ of 41.1 μM and 3.68 μM, respectively) (Mitcheson and Hancox, 1997; Terrenoire et al., 2013; Du et al., 2014; Gualdani et al., 2015; Figures 2B–E, Supplementary Figures 2A,B). The non-selective β-adrenergic agonist, isoproterenol, as well as the β2-selective agonist, formoterol, both induced positive chronotropic responses at expected doses (EC_50_ of 18 nM and ~2.6 nM) (Wills et al., 2012; Sirenko et al., 2013; Figures 2F–I, Supplementary Figures 2C,D). The non-selective β-adrenergic antagonist, propranolol, prolonged the APD as well as slowed the beat rate considerably also as previously reported (EC_50_ of 51.7 μM) (Sirenko et al., 2013; Figures 2J,K, Supplementary Figure 2E). The L-type calcium channel inhibitors verapamil and isradipine shortened the APD as expected and increased the beat rate (EC_50_ of ~8.06 μM and ~232 nM) (Figures 2l–O, Supplementary Figures 2F,G) while the L-type calcium channel agonist, BayK 8644, significantly prolonged the APD (EC_50_ of ~68.5nM) (Figures 2P,Q, Supplementary Figure 2H). To evaluate the presence and functionality of sodium channels in the hiPSC-CMs, the cells were treated with specific sodium channel inhibitor, tetrodotoxin (TTX), and two sodium channel agonists, anemonia sulcata toxin (ATX-II) and veratridine, which bind to different sites on the sodium channel and open the channel by slightly different mechanisms (Stevens et al., 2011). TTX caused a cessation of beating (EC_50_ of 4.96 μM) at doses comparable to the IC_50_ for peak sodium current (I_NaP_) inhibition in CMs (>1 μM) indicating a reliance on sodium channel currents for the generation of APs (Figures 2R,S, Supplementary Figure 2I). However, dependence of the AP on sodium current varied across batches of CMs, suggesting inconsistency in the maturity of the hiPSC-CMs. The two sodium channel agonists prolonged the APD without inducing EADs (426 nM for veratridine and 198 nM for ATX-II) (Figures 2T–W, Supplementary Figures 2J,K), possibly reflecting either the maturation state of the hiPSC-CMs or genetic variation in the hiPSCs from different individuals.

To assess cross-platform reproducibility, we tested TTX, isoproterenol and E-4031 using Molecular Devices ImageXpress Micro XLS (Supplementary Figure 3, Supplementary Movies 2, 3) using iPSC-CMs prepared in-house (Cunningham et al., 2017), revealing similar dose responsiveness (EC_50_ for isoproterenol 77 nM, EC_50_ for TTX 4.85 μM, EC_50_ for E-4031 >13.1 nM, EC_50_ for ATX-II 1.03 μM). In summary, optical recording of hiPSC-CM AP waveform kinetics in response to reference compounds reveals the expected functional expression of cardiac ion channels (see Supplementary Table 2).

### Automated quantification of drug-induced arrhythmias

**Figure 3:**
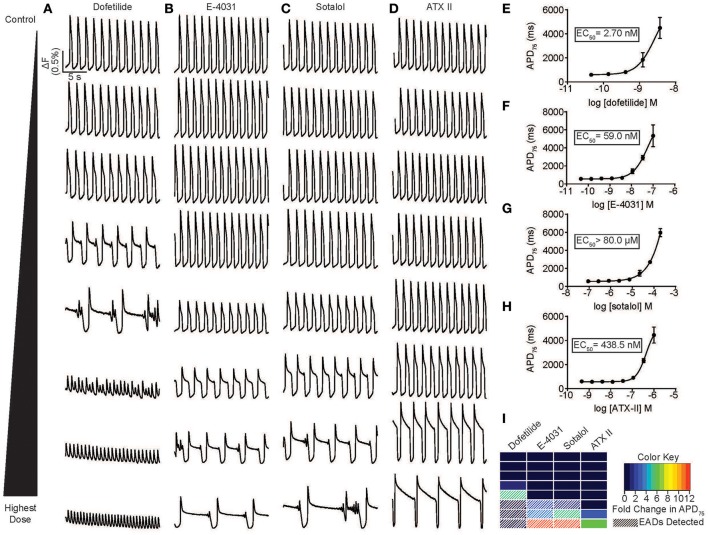
Automated high throughput characterization of drug-induced arrhythmias. **(A–D)** Representative ΔF/F_0_ vs. time plots (33 Hz) for a dose response with dofetilide (0.1–100 nM) **(A)**, E-4031 (0.1–100 nM) **(B)**, sotalol (0.2 –200 μM) **(C)**, and ATX-II (1–1,000 nM) **(D)** in normal individual (iCell) hiPSC-CMs. **(E–H)** Dose response curve for APD_75_ for dofetilide **(A)**, E-4031 **(B)**, sotalol **(C)**, and ATX-II illustrating a dose dependent prolongation of the APD and an EC_50_ values of 2.7, 59 nM, >80 and >200 μM, respectively. Each point represents an individual well, *n* = 3. Error bars are s.e.m. **(I)** A heat map illustration the magnitude of prolongation and automated detection of EADs for an 8-point dose range for dofetilide, E-4031, sotalol, and ATX-II. Automated detection of EADs signified by white stripes. Scale bars indicate time (5 s) and ΔF (0.5% of the value in each well).

The appearance of EADs and voltage alternans are the cellular manifestations of T-wave prolongation and PVCs seen on the ECG that predispose patients to VT and TdP. Therefore, we analyzed the incidence of EADs in hiPSC-CMs treated with inhibitors of I_Kr_ and an agonist of the late sodium current (I_NaL_). The development of these morphologies can be better assessed over longer periods of recording (Mohammad et al., 1997; Song et al., 2008). Thus, time series images were acquired for 20 s at 33 Hz to provide improved signal to noise as well as recording duration, and coupled with an automated image analysis to determine the effect of the hERG blockers dofetilide, E-4031 and sotalol. All three molecules caused extensive dose-dependent prolongation of APD, induction of EADs and, at some doses, voltage alternans (Figures 3A–C, see also Supplementary Figure 3). Representative movie clips for dofetilide illustrate the presence of EADs as compared to untreated hiPSC-CMs (Supplementary Movies 4, 5), and treatment with 33 and 100 nM dofetilide caused rapidly firing subthreshold APs that did not induce contraction (Supplementary Movies 6, 7). The appearance of these AP morphologies seemed analogous to tachyarrhythmia or fibrillation. To determine an EC_50_ dose for prolongation of the APD, dose response curves were generated from the APD_75_ values (Figures 3E–H, see also Supplementary Figure 3) in addition to the automatic detection of EADs (Figure 3I). Each of the hERG inhibitors induced significant APD prolongation as well as induced EADs at expected EC_50_ values (2.79 nM for dofetilide, 59.0 nM for E-4031 and ~80 μM for sotalol). Interestingly, EADs were not detected in the ATX-II condition in the CDI hiPSC-CMs, although the compound prolonged the APD more than 7-fold (EC_50_ of 438.5 nM), which was comparable to the extent of prolongation by dofetilide and sotalol. However, we have noted EADs in other hiPSC-CM populations, most likely reflecting cell line or differentiation protocol-specific differences (Supplementary Figure 3D). In conclusion, optical recording visualized drug induced prolongation of the APD, induction of EADs as well as tachyarrhythmia *in vitro*.

### Reversion of congenital and drug induced APD prolongation

**Figure 4:**
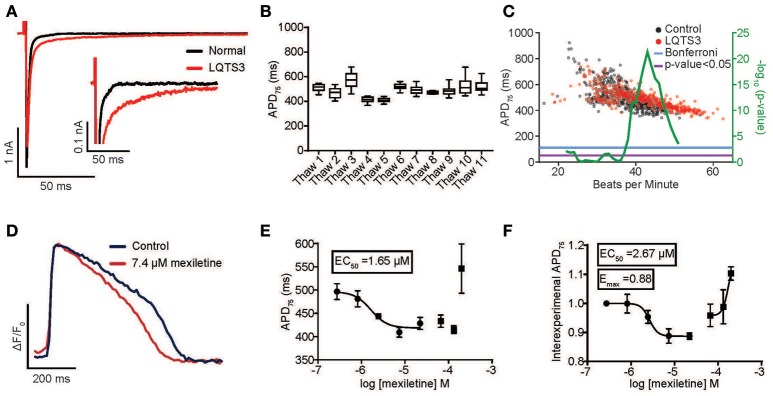
Reversion of congenital long QT syndrome type 3 in hiPSC-CMs. **(A)** Sodium current recording from an LQTS3 (F1473C) hiPSC-CM (red trace) exhibiting increase late sodium current in response to a 150 ms voltage step from –80 to –40 mV as compared to a normal individual (iCell) hiPSC-CM (black trace). I_NaL_ was 1.2% ± 0.604 of I_NaP_ in the LQTS3 hiPSC-CMs for *n* = 10. There was no detectable late current in the normal hiPSC-CMs. **(B)** Average APD_75_ from controls wells for all LQTS3 hiPSC-CM experiments plotted for multiple thaws of the same lot. **(C)** Plot of Average APD_75_ vs. BPM from individual LQTS3 and normal cardiomyocyte wells. Mann-Whitney test using a sliding window (center value ± 3) (green) with Mann-Whitney *p* < 0.05 (purple) and Bonferroni corrected *p*-value (blue). **(D)** An average normalized ΔF/F_0_ vs. time plot (100 Hz) from all peaks each well (*n* = 3) either untreated or with treated 7.4 μM mexiletine in LQTS3 hiPSC-CMs. Scale bars indicate time (200 ms) and ΔF/F_0_ (50% of the maximal obtained in each well). **(E)** A dose response curve for APD_75_ for a single experiment with LQTS3 hiPSC-CMs. EC_50_ value is 1.65 μM. Each point represents an individual well, *n* = 3. Error bars are s.e.m. **(F)** A dose response curve from multiple thaws of LQTS3 hiPSC-CMs (*n* = 4). Each point represents the average ± s.e.m for *n* = 4 separate experiments of the normalized change in APD_75_ for each dose of mexiletine from all experiments.

Congenital and pharmacological hiPSC-CM models of arrhythmia are of enormous interest as tools to decipher arrhythmogenic mechanisms. Moreover, an individual's genetic makeup (particularly polymorphisms in voltage-gated ion channels) can predispose patients to drug-induced arrhythmia and might underlie the sporadic nature of this phenomenon in the population (Yang et al., 2002). Therefore, visualizing drug-induced arrhythmia in hiPSC-CM models from healthy and diseased individuals is potentially a powerful new tool to personalize treatment as well as to predict liability of drug candidates across “high risk” individuals. To test this hypothesis, we evaluated a previously characterized LQTS3 patient harboring a F1473C missense mutation in the cardiac sodium channel, Na_v_1.5 (Terrenoire et al., 2013). Whole cell patch clamp recording of hiPSC-CMs derived from the LQTS3 patient confirmed increased I_NaL_ (I_NaL_ was 1.2% ± 0.604 of I_NaP_ in the LQTS3 hiPSC-CMs, *n* = 10, and no detectable late current in the normal hiPSC-CMs) (Figure 4A, Supplementary Movie 8), which is a hallmark of LQTS3, indicating that the cells were a bona fide model of the congenital channelopathy. We used the high throughput platform to evaluate the reproducibility of APD across multiple preparations of LQTS3 hiPSC-CMs. Intra-experimental APDs of spontaneously beating hiPSC-CMs were very consistent with an average coefficient of variation (CV) = 0.069 ± 0.019 for untreated wells across multiple plates from the same thaw. The inter-experimental average APD from vials thawed on different days had more variability (CV = 0.098) (Figure 4B). We plotted the average APD for every untreated, control well across all experiments and a statistically significant difference in APD between the normal and LQTS3 hiPSC-CMs was discernible only above a spontaneous beat rate of 37 beats per minute (BPM) based on the Bonferroni corrected *p* < 0.05 (Figure 4C) which is consistent with prior studies showing a phenotypic variability of the LQTS3 phenotype (Ma et al., 2013; Paci et al., in press). Nonetheless, when probed pharmacologically with mexiletine, a sodium channel inhibitor with selectivity for I_NaL_ over peak sodium current (I_NaP_) (Wang et al., 1997), LQTS3 hiPSC-CMs showed a stereotypic, dose-dependent AP shortening, effectively reversing the LQTS3 phenotype (EC_50_ = 1.65 μM) (Figures 4D,E). This reversion was highly consistent across 4 different thaws of LQTS3 hiPSC-CMs spanning 38–53 BPM, as seen when baseline APD is normalized for each dose (average EC_50_ = 2.67 μM and average E_max_ of 0.88) indicating a robust reversion of the disease phenotype that was independent of the variation in APD and beat rate (Figure 4F).

**Figure 5:**
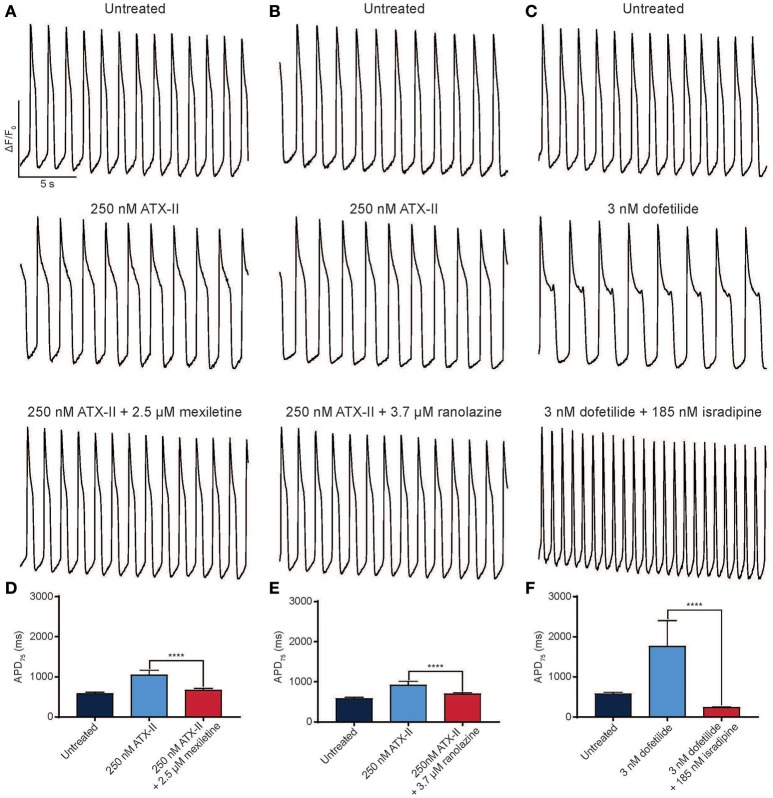
Reversion of drug-induced arrhythmia with antiarrhythmic drugs. **(A–C)** Representative normalized ΔF/F_0_ vs. time plots for normal individual (iCell) hiPSC-CMs treated with 250 nM ATX-II and 2.5 μM mexiletine **(A)** or 3.7 μM ranolazine **(B)** and 3 nM dofetilide and 185 nM isradipine **(C)**. **(D–F)** Quantification of APD_75_ for normal hiPSC-CMs treated with ATX-II and mexiletine **(D)**, ATX-II and ranolazine **(E)**, dofetilide and isradipine **(F)**. ****p < 0.0001 using a one-way ANOVA test. *n* = 3 replicate wells for each condition in all cases. Scale bars indicate time (5 s) and ΔF/F_0_ (50% of the maximal obtained in each well).

Pharmacological models of LQTS are commonly used to probe drug effects on APD and arrhythmia (Shimizu and Antzelevitch, 1999). LQTS3 can be modeled pharmacologically by treatment with ATX-II, which phenocopies the disease by specifically inhibiting fast inactivation of cardiac sodium channels. ATX-II-induced APD prolongation can be effectively reverted by 2.5 μM mexiletine (Figures 5A,D) and another I_NaL_ selective inhibitor ranolazine (3.7 μM) (Figures 5B,E). Finally, we created a pharmacological model of LQTS2 using the I_Kr_ inhibitor, dofetilide. APD prolongation caused by 3 nM dofetilide was effectively reverted by 185 nM isradipine, an L-type calcium channel inhibitor (Figures 5C,F) (as expected because blocking the inward calcium current counteracts the block to the outward potassium current). Thus, the automated platform facilitates the characterization of diseased hiPSC-CMs and pharmacological models of disease in normal cells, and in both cases, can detect the therapeutic effect of small molecule drugs.

## Discussion

Using an optical method for high throughput detection of AP kinetics, we developed an automated platform for assessment of proarrhythmia in normal and channelopathy patient derived hiPSC-CMs. Our optimized procedures can be readily implemented on automated microscopes and provides a cost effect alternative to microelectrode arrays (MEAs) with the added benefit of detecting action potentials directly rather than inferring it from extracellular field potentials. Although other methods for the quantification of arrhythmia in hiPSC-CMs have been used previously to record the electrophysiological effects of reference compounds including MEAs (Guo et al., 2011; Navarrete et al., 2013; Gilchrist et al., 2015), optogenetic methods (Dempsey et al., 2016; Klimas et al., 2016), calcium sensitive probes (Sirenko et al., 2013; Pfeiffer et al., 2016) and VSPs (Lopez-Izquierdo et al., 2014; Bedut et al., 2016; Zeng et al., 2016), our study is the first large scale, automated evaluation of reference compounds in 384 well plate format using patient-derived hiPSC-CMs by any modality, and facilitates the rapid generation of dose response curves for many physiologically relevant parameters. Moreover, our study is the first to use a VSP to characterize a patient-specific hiPSC-CM disease model using an automated platform to quantify arrhythmia phenotypes and demonstrate reversion of those disease phenotypes with small molecule therapeutics *in vitro*.

The hiPSC-CMs presented arrhythmia phenotypes (voltage alternans, EADs, rapid APs resembling fibrillation, and cessation of beating) in response to reference compounds at expected doses. These results are consistent with previous studies indicating that hiPSC-CMs can discriminate the proarrhythmic influences of small molecules and inherited channel mutations (Matsa et al., 2011; Liang et al., 2013). By developing an assay in 384-well plate format and incorporating optical recording with automated analyses, our study advances the use of hiPSC-CMs as tools for the evaluation of arrhythmogenic potential of candidate molecules that complement conventional, lower throughput assays, such as whole animals or *ex vivo* heart preparations. In addition, hiPSC-CM technology allows individual patient backgrounds to be evaluated with greater throughput than can be achieved with conventional methods. The increased throughput enables unbiased functional genomics screening of proteins or genes (i.e., siRNAs, miRNAs or CRISPR gene knockouts) in diverse genetic backgrounds of normal and CHD patient-specific hiPSC-CMs, accelerating our ability to generate systems level insight into arrhythmia mechanisms.

Important limitations in the use of hiPSC-CMs to evaluate pro-arrhythmia include differences in ion channel physiology between these cells and adult cardiomyocytes, such as a larger I_f_ that confers automaticity, and reduced I_K1_ that is involved in maintaining the low resting potential of adult CMs (Del Alamo et al., 2016). Although the relatively pure and synchronously contracting monolayer hiPSC-CM cultures allow us to discern cellular arrhythmia phenotypes in the 384-well format, these cultures cannot reliably reproduce arrhythmia mechanisms that depend on complex 3D architecture of the normal or diseased heart. Additionally, we found that the ability to discern chamber specific AP waveforms as well as chamber specific drug effects required the culture of pure populations of sub type specific CMs and could not be identified through single cell analysis of our synchronously contracting monolayers. hiPSC-CM differentiation and culture methods are advancing, and recent nano and micro patterning of cell culture substrates have already improved the fidelity of the hiPSC-CMs as models of adult cardiomyocyte physiology, in particular calcium-induced calcium release, force-generation and adrenergic signaling (Kim et al., 2010; Feaster et al., 2015; Ribeiro A. J. et al., 2015; Jung et al., 2016). Such advances would improve the ability to correlate the cellular arrhythmia phenotypes recorded here with clinical arrhythmia. Key areas for further improvement include sarcomeric structural organization (Pasqualini et al., 2015) force generation (Ribeiro A. J. et al., 2015; Ribeiro M. C. et al., 2015), metabolism (Yang et al., 2014) as well as chamber subtype specific differentiation protocols (Devalla et al., 2015). In addition, while performing this study, we observed that certain hiPSC-CM batches (and differentiation protocols) yielded cells that were resistant to TTX-induced cessation of beating at the time the cells were tested. Moreover, CMs from some hiPSC lines presented EADs in response to ATX-II (generated in-house) whereas others (i.e., iCell) did not (Figure 3D and Supplementary Figure 3D). Such variability might reflect differences in cell donors or CM differentiation protocols. TTX-resistant batches responded to ATX-II by AP prolongation, indicating that sodium channels are typically present but are inactive consistent with a relatively depolarized diastolic membrane potential (e.g., Veerman et al., 2015). Given the variability in maturation state of cardiomyocytes due to differences in time in culture as well as the protocol used to differentiate cardiomyocytes from hiPSCs, AP dependence on sodium channels should be evaluated across protocols and commercial hiPSC-CM preparations. Finally, our protocol and instrument parameters did not evaluate AP resting potential or V_max_ because of non-ratiometric nature of the VSP and the acquisition frame rate (100 frames/s), respectively, although a faster frame rate is feasible on the IC200.

In summary, hiPSC-CM technology in cardiovascular drug discovery and clinical management of heart disease is emerging as a viable paradigm. Indeed, low throughput methods have shown that it is possible to revert arrhythmia phenotypes in hiPSC-CM channelopathy models following drug treatment, and that the influence of patient genetic makeup on drug-induced arrhythmia is manifest in the cell cultures (Sharma et al., 2013). Automated 384-well plate screening enables large-scale studies that should make it possible to introduce testing of patient cells at early stages of drug development, decipher basic arrhythmia mechanisms, and test whether individual patient differences can predict clinical outcome.

## Author contributions

WM, AS, MY, AC, FC, and AB conducted the experiments and analyzed the data. EM synthesized VF2.1.Cl. JP, JC, and MM obtained funding. WM and MM wrote, and everyone edited, the manuscript.

### Conflict of interest statement

JP is a founder and MM is on the scientific advisory board of Vala Sciences, and both have equity in the company, which manufactures a high content instrument used in these studies. The other author declares that the research was conducted in the absence of any commercial or financial relationships that could be construed as a potential conflict of interest. The reviewer SC and handling Editor declared their shared affiliation, and the handling Editor states that the process nevertheless met the standards of a fair and objective review.
