# Supplementary material for: An Automated Platform for Assessment of Congenital and Drug-Induced Arrhythmia with hiPSC-Derived Cardiomyocytes
Source: Front Physiol. 2017 Oct 11;8:766. doi: 10.3389/fphys.2017.00766 (PMC5641590; doi:10.3389/fphys.2017.00766)
Supplement: Supplementary file 9 [file DataSheet1.pdf]

Supplementary Material

An Automated Platform for Assessment of Congenital and Drug-Induced Arrhythmia with hiPSC-derived Cardiomyocytes

Wesley L. McKeithan, Alex Savchenko, Michael S. Yu, Fabio Cerignoli, Arne A. N. Bruyneel, Jeffery H. Price, Alexandre R. Colas, Evan W. Miller, John R. Cashman and Mark Mercola

Contents

Supplementary Figure 1

Supplementary Figure 2

Supplementary Figure 3

Supplementary Table 1

Supplementary Table 2

Supplementary Movie Legends

References

Supplementary Figure 1: Optimization of VF2.1.Cl imaging and analysis

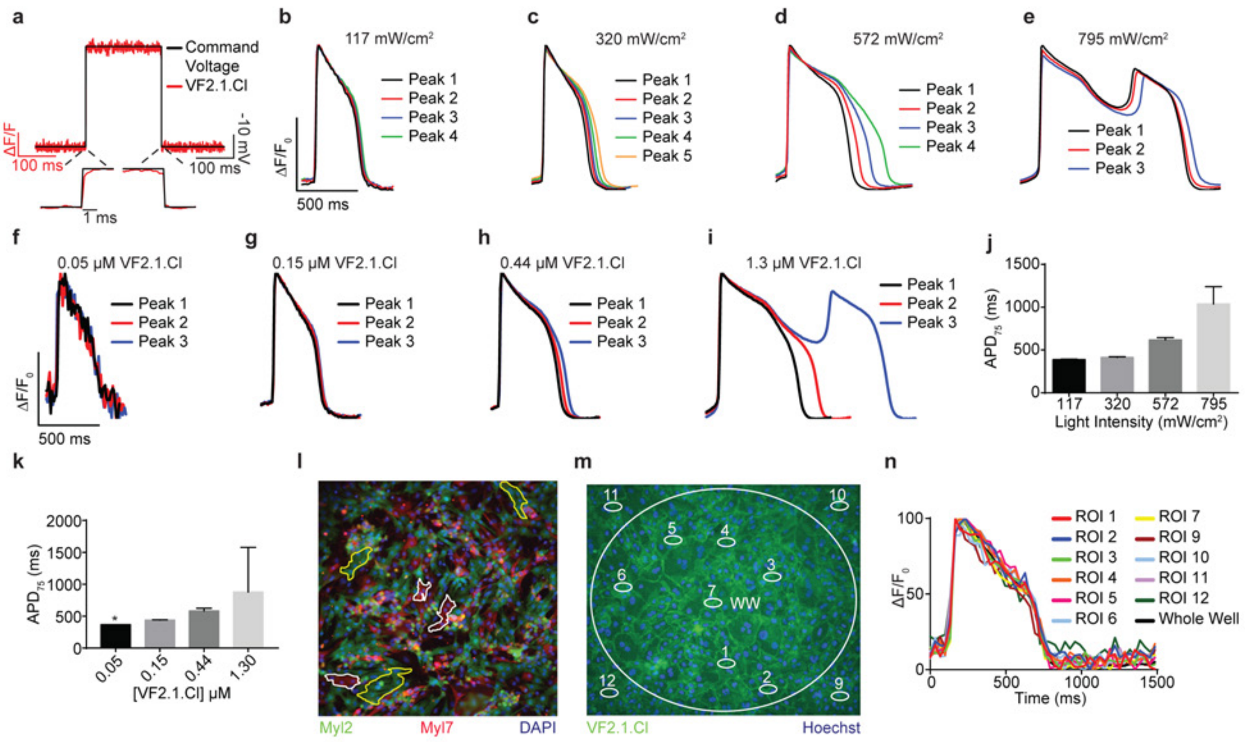

(**a**) Response time of VF2.1.Cl in response to a depolarizing pulse from -90 mV to -40 mV recorded with a photomultiplier tube with recording frequency of 10 kHz. The inset shows both the rise and decay over 5 ms.

(**b-i**) Normalized ΔF/F vs. time for hiPSC-CMs with 200 nM VF2.1.Cl in response to increasing light intensity (**b-e**) or with 117 mW/cm^2 (485/20 nm) of light in response to increasing concentrations of VF2.1.Cl (**f-i**). The peak numbers in the legend correspond to consecutive peaks from a single well over 6 seconds.

(**j,k**) Average APD_{75} for consecutive peaks in a peak train from a single well loaded with 200nM VF2.1.Cl in response to increasing amounts light intensity (485/20 nm) (**j**) or train from a single well illuminated with 117 mW/cm^2 in response to increasing concentrations of VF.2.1.Cl (mean ± SD).

(**l**) Immunofluorescence staining for Myl2 (ventricular specific marker) and Myl7 (atrial specific marker) in culture of 1:1 mixed neonatal rat atrial and ventricular CMs. Regions of interest (ROIs) used to generate action potential traces for groups of atrial CMs (white) and ventricular CMs (yellow).

(**m**) Image with ROIs for hiPSC-CMs loaded with VF.2.1.Cl and Hoechst 33258 to generate ΔF/F plots. WW indicates the whole well ROI.

(**n**) Overlay of ΔF/F vs. time plots generated from ROIs in hiPSC-CMs from (**m**). * indicates only a single peak was automatically detected because of low signal to noise.

Supplementary Figure 2: Reference compound dose response curves

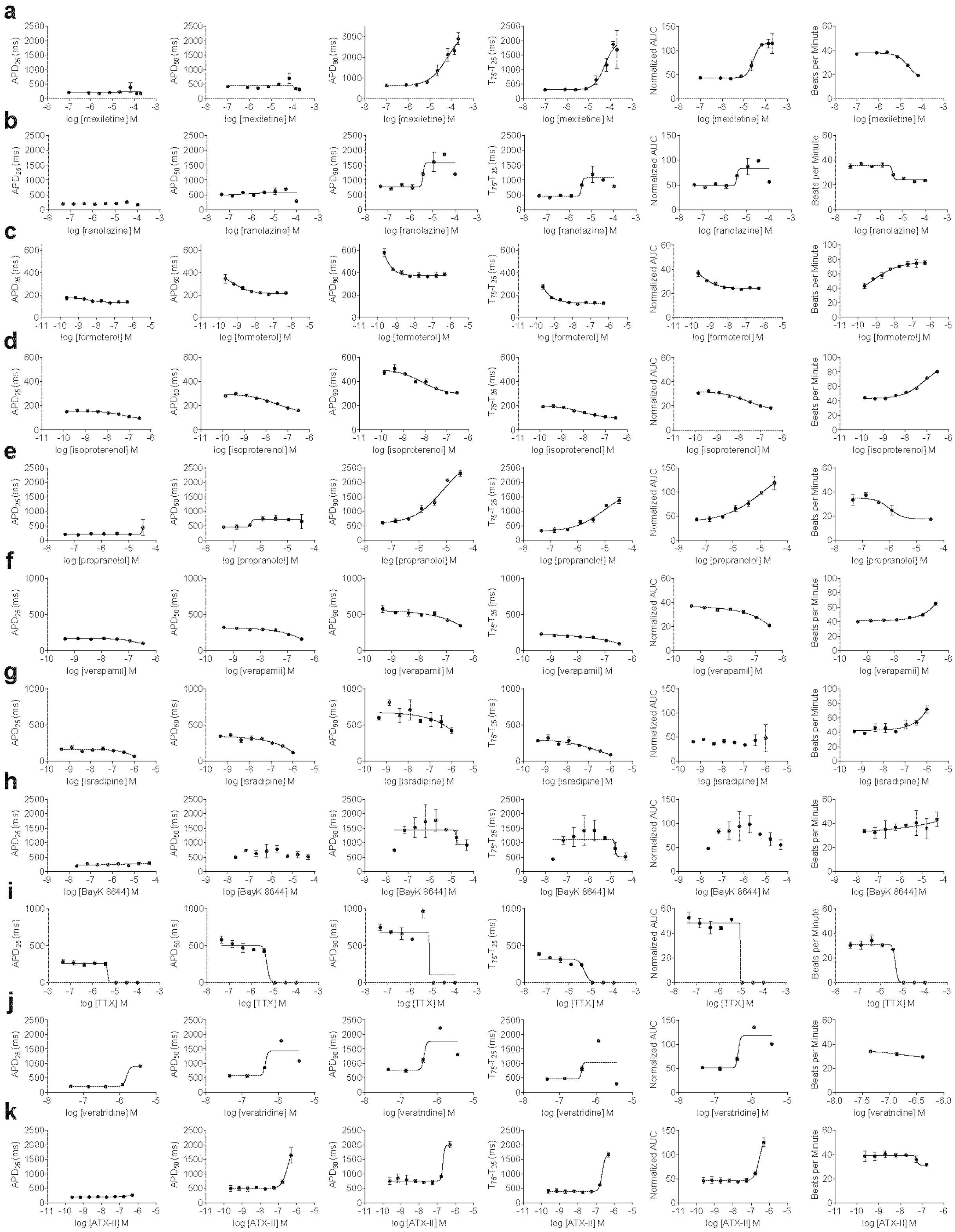

(**a-j**) Dose response curves for APD_{75}, APD_{25}, APD_{50}, APD_{90}, $Time_{75}$-$Time_{25}$, Normalized area under the curve (AUC) and Beats per minute for (**a**) mexiletine, (**b**) isoproterenol, (**c**) formoterol, (**d**) propranolol, (**e**) verapamil, (**f**) isradipine, (**g**) BayK 8644, (**h**) TTX, (**i**) veratridine and (**j**) ATX-II

Supplementary Figure 3: Cross-platform reproducibility of optimized conditions using the ImageXpress Micro XLS

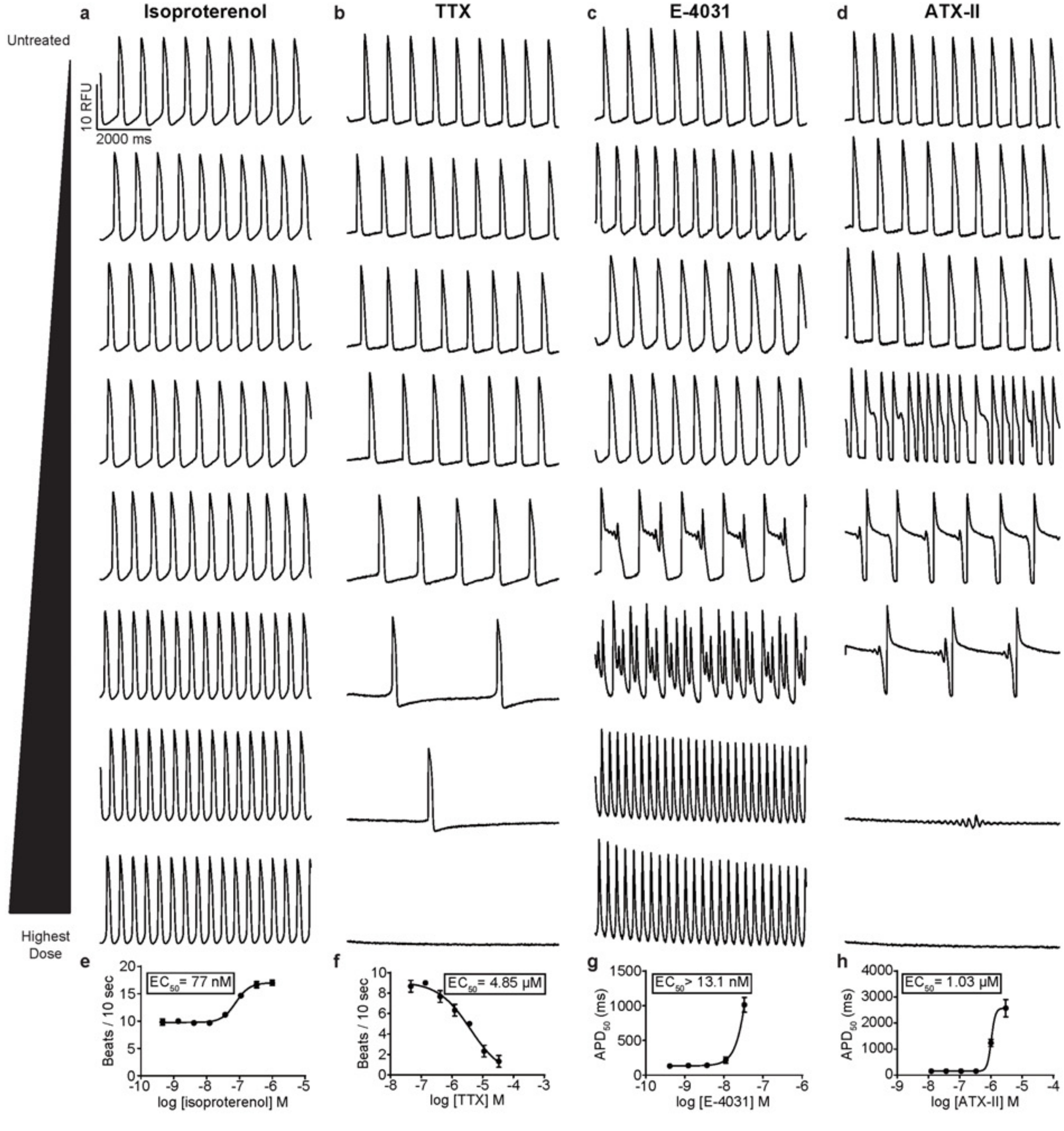

(**a-d**) Representative ΔF/F vs. time plots for a dose response with isoproterenol (1.2 nM-1000 nM) (**a**), TTX (0.1 μM-100 μM) (**b**), E-4031 (1.2 nM-900 nM) (**c**), and ATX-II (37 nM-27 μM) (**d**) in hiPSC-CMs using the Molecular Devices ImageXpress Micro XLS imaging system.

(**e-h**) Dose response curve for beat rate for isoproterenol (**e**) and TTX (**f**) as well as dose response curve for APD_{50} for E-4031 (**g**) and ATX-II (**h**). Each point represents an individual well, n = 3. Error bars are s.e.m.

Supplementary Table 1: hiPSC-CM lot numbers and purity

Cardiomyocyte ID	% Cardiomyocytes
iCell Lot: 1097546	98 % (reported on CoA)
iCell Lot: 1291715	99 % (reported on CoA)
iCell Lot: 1291715	98 % (reported on CoA)
myCell LQTS3 Lot: 01583.763.CM001	97 % (reported on CoA)
hiPSC (SCVI15)-Cardiomyocytes Lot: iPSC-Id1(0424)-20160609 and iPSC-Id1(8/23)-20161004	>80% (α-actinin +)

Supplementary Table 2: Comparison of human C_{max} values with changes in AP kinetics

Drug	C_{max} (μM)	EC_{50} (μM)
sotalol	9.39 (Leahey et al., 1980)	> 80
dofetilide	0.0089 (Le Coz et al., 1995)	0.0027
mexiletine	3.43 (Klein et al., 1985)	41 (prolongation in normal patient hiPSC-CMs)<br>1.65 (shortening in LQTS3 patient hiPSC-CMs)
ranolazine	2-6 (Chaitman, 2006)	3.68
isoproterenol	0.0076 (Reyes et al., 1993)	0.018
propranolol	0.672 (Wilson et al., 1982)	51.7
verapamil	0.584 (McCourty et al., 1988)	> 8.06
israpidine	0.016 (Shenfield et al., 1990)	> 232

Supplementary Movie Legends

Supplementary Movie 1: Normal (healthy individual iCell) hiPSC-CMs recorded at 100 Hz with VF2.1.Cl using the IC200 KIC at 20x magnification. The movie is played back in real time.

Supplementary Movie 2: Untreated normal (SCVI15) hiPSC-CMs recorded at 100Hz with VF2.1.Cl using the ImageXpress at 10x magnification. The movie is played back in real time.

Supplementary Movie 3: Normal (SCVI15) hiPSC-CMs treated with 1 µM isoproterenol recorded at 100Hz with VF2.1.Cl using the ImageXpress at 10x magnification. The movie is played back in real time.

Supplementary Movie 4: untreated normal (healthy individual iCell) hiPSC-CMs recorded at 33 Hz for 20 seconds with the IC200 KIC at 20x magnification. The movie is played back in real time.

Supplementary Movie 5: Normal (healthy individual iCell) hiPSC-CMs treated with 3.7 nM dofetilide recorded at 33 Hz for 20 seconds with the IC200 KIC at 20x magnification. The movie is played back in real time.

Supplementary Movie 6: Normal (healthy individual iCell) hiPSC-CMs treated with 11 nM dofetilide recorded at 33 Hz for 20 seconds with the IC200 KIC at 20x magnification. The movie is played back in real time.

Supplementary Movie 7: Normal (healthy individual iCell) patient hiPSC-CMs treated with 100 nM dofetilide recorded at 33 Hz for 20 seconds with the IC200 KIC at 20x magnification. The movie is played back in real time.

Supplementary Movie 8: LQTS3 (F1473C MyCell) hiPSC-CMs recorded at 100 Hz with VF2.1.Cl using the IC200 KIC at 20x magnification. The movie is played back in real time.

References

Chaitman, B.R. (2006). Ranolazine for the treatment of chronic angina and potential use in other cardiovascular conditions. *Circulation* 113, 2462-2472.

Klein, A., Sami, M., and Selinger, K. (1985). Mexiletine kinetics in healthy subjects taking cimetidine. *Clin Pharmacol Ther* 37, 669-673.

Le Coz, F., Funck-Brentano, C., Morell, T., Ghadanfar, M.M., and Jaillon, P. (1995). Pharmacokinetic and pharmacodynamic modeling of the effects of oral and intravenous administrations of dofetilide on ventricular repolarization. *Clin Pharmacol Ther* 57, 533-542.

Leahey, W.J., Neill, J.D., Varma, M.P., and Shanks, R.G. (1980). Comparison of the activity and plasma levels of oxprenolol, slow release oxprenolol, long acting propranolol and sotalol. *Eur J Clin Pharmacol* 17, 419-424.

Mccourty, J.C., Silas, J.H., Tucker, G.T., and Lennard, M.S. (1988). The effect of combined therapy on the pharmacokinetics and pharmacodynamics of verapamil and propranolol in patients with angina pectoris. *Br J Clin Pharmacol* 25, 349-357.

Reyes, G., Schwartz, P.H., Newth, C.J., and Eldadah, M.K. (1993). The pharmacokinetics of isoproterenol in critically ill pediatric patients. *J Clin Pharmacol* 33, 29-34.

Shenfield, G.M., Boutagy, J., Stokes, G.S., Rumble, F., and Dunagan, F. (1990). The pharmokinetics of isradipine in hypertensive subjects. *Eur J Clin Pharmacol* 38, 209-211.

Wilson, T.W., Firor, W.B., Johnson, G.E., Holmes, G.I., Tsianco, M.C., Huber, P.B., and Davies, R.O. (1982). Timolol and propranolol: bioavailability, plasma concentrations, and beta blockade. *Clin Pharmacol Ther* 32, 676-685.
